# Discovery of a novel RSK2 inhibitor for the treatment of metastatic pancreatic cancer

**DOI:** 10.1080/14756366.2025.2538673

**Published:** 2025-08-05

**Authors:** Chi-Hsiu Chung, Kai-Cheng Hsu, Ming-Min Huang, Huang-Ju Tu, Shiow-Lin Pan, Min-Wu Chao

**Affiliations:** ^a^ School of Medicine, College of Medicine, National Sun Yat-sen University, Kaohsiung, Taiwan; ^b^ Department of Medical Education and Research, Kaohsiung Veterans General Hospital, Kaohsiung, Taiwan; ^c^ Department of Dermatology, School of Medicine, National Yang Ming Chiao Tung University, Taipei City, Taiwan; ^d^ Graduate Institute of Cancer Biology and Drug Discovery, College of Medical Science and Technology, Taipei Medical University, Taipei, Taiwan; ^e^ Ph.D. Program for Cancer Molecular Biology and Drug Discovery, College of Medical Science and Technology, Taipei Medical University, Taipei, Taiwan; ^f^ TMU Research Center of Cancer Translational Medicine, Taipei Medical University, Taipei, Taiwan; ^g^ Institute of Biopharmaceutical Sciences, College of Medicine, National Sun Yat-sen University, Kaohsiung, Taiwan; ^h^ Institute of Precision Medicine, College of Medicine, National Sun Yat-sen University, Kaohsiung, Taiwan

**Keywords:** RSK2 inhibitor, pancreatic cancer, metastasis, structure-based virtual screening

## Abstract

Pancreatic cancer is among the most lethal malignancies, with a five-year survival rate of only 6%. For patients with metastatic disease, current treatments extend median survival by merely four months. This study addresses the urgent need for targeted therapies, as no specific drugs are currently available. Clinical analyses revealed significantly elevated RSK2 expression in pancreatic cancer tissues, associated with shorter survival. We aimed to identify a novel RSK2 inhibitor for metastatic pancreatic cancer. Through structure-based virtual screening, we identified NSYSU-115 as a promising candidate with an IC50 of 45.5 nM. At low concentrations, NSYSU-115 significantly suppressed colony formation, while higher concentrations reduced cell viability and proliferation. It also inhibited phosphorylation of IκBα, a known RSK2 substrate, in a dose- and time-dependent manner. Furthermore, NSYSU-115 impaired cell migration and altered epithelial-mesenchymal transition (EMT) markers. These findings highlight NSYSU-115 as a potent kinase inhibitor with promising therapeutic potential for pancreatic cancer treatment.

## Introduction

Pancreatic cancer (PC) is among the deadliest malignancies, with a 5-year survival rate of just 6%^1–3^. For resectable cases, gemcitabine can improve survival to 21%, but metastatic PC remains particularly challenging, with a median survival of only 6.8 months despite treatments like FOLFIRINOX providing limited benefits^4–6^. Due to the absence of early symptoms, PC is often diagnosed at advanced stages, where it genetic and phenotypic heterogeneity further complicates treatment efficacy^7^. Based on RNA profiles, PC is categorised into subtypes: the basal subtype (aggressive, poor prognosis) and the classical subtype (better prognosis), with some tumours exhibiting mixed features^8^. The basal subtype, associated with higher metastasis and mortality, highlights the urgent need for targeted therapies, as no effective drugs currently exist for metastatic PC.

Pancreatic ductal adenocarcinoma (PDAC), commonly referred to as “pancreatic cancer,” serves as a prototype for similar adenocarcinomas in the biliary tract, ampullary region, and gallbladder, sharing common morphology and behaviour^9^. Over 90% of PDAC cases harbours mutations in the v-Ki-ras2 Kirsten rat sarcoma viral oncogene homolog (KRAS), a critical genetic factor. The genomic landscape of PDAC also includes alterations in key tumour suppressor genes that regulate cell growth and genomic stability^10^. KRAS activation is observed in precursor lesions such as pancreatic intraepithelial neoplasia (PanIN) and intraductal papillary mucinous neoplasm (IPMN), identifying it as a major genetic driver^11^. Unfortunately, despite decades of research, KRAS mutations have remained largely untreatable due to the challenges in developing effective KRAS-targeted drugs.

The p90 ribosomal S6 kinase (RSK) family, a downstream effector in the Ras/Raf/MEK/ERK signalling pathway, includes closely related serine/threonine kinases that regulate diverse cellular processes^12^. While some RSK family members promote tumour growth, others exhibit tumour-suppressive properties^13^^,^^14^. Among them, RSK2, has been implicated in cancer cell metastasis, with studies showing that RSK2 deletion reduces metastatic potential in xenograft models^15^. Pharmacological inhibition of RSK2 has been shown to suppress cell proliferation, migration, invasion, and metastasis in various cancer types^16^. Studies have demonstrated that RSK2, when activated by hypoxia or ERK signalling, influences cancer cell motility and invasion through multiple pathways^17^, including activating EMT-associated genes, modulating integrin activity, and reorganising the actin cytoskeleton. Mechanistically, RSK2 phosphorylates IκBα, facilitating NF-κB entry into the nucleus and promoting the expression of genes involved in cytokine secretion, cancer migration, and invasion^18^. These findings suggest that an RSK2 inhibitor holds significant potential for managing pancreatic cancer metastasis.

Pancreatic cancer (PC) presents substantial treatment challenges, with low median survival rates following metastasis, underscoring the urgent need for innovative therapeutic strategies. Among potential molecular targets, RSK2 has emerged as a key regulator of cell migration and tumour progression. This research seeks to address this critical gap by identifying a novel compound that specifically targets RSK2. Using the National Cancer Institute (NCI) compound database, which comprises approximately 280,000 compounds, we employed structure-based virtual screening to identify small molecules with inhibitory effects on RSK2. Candidate compounds were selected and validated through enzymatic assays, followed by structure-activity relationship (SAR) analysis. The most potent compound was further assessed for cytotoxicity, protein phosphorylation, migration inhibition, and alterations in epithelial-mesenchymal transition (EMT) markers *in vitro*. In conclusion, this study highlights NSYSU-115 as a structurally novel and promising kinase inhibitor with therapeutic potential for PC, offering prospects for reducing metastatic progression.

## Materials and methods

### Structure-based virtual screening

A structure-based virtual screening was performed to identify potential RSK2 inhibitors. The compound library, consisting of approximately 280,000 compounds, was obtained from the National Cancer Institute (NCI). The compound set was first refined by removing those containing Pan-Assay Interference Compounds (PAINS) structures^19^. Compounds with a Quantitative Estimate of Drug-Likeness (QED) score ≤ 0.25 were also excluded^20^. Next, the remaining compounds were filtered based on Lipinski’s and Veber’s rules. The RSK2 structure (PDB ID: 5D9L) was retrieved from the Protein Data Bank (PDB) ^21^, and both the protein and compound structures were prepared using Maestro^22^. The co-crystal ligand (583) is an RSK inhibitor complexed in the RSK2 structure (PDB ID: 5D9L), which was used to identify the binding site^23^. Compounds were then docked into the binding site using Glide^24^. The docked compounds were ranked by their docking scores, and the top-ranked compounds were selected for further analysis. A common feature of kinase inhibitors targeting this binding site is the formation of hydrogen bonds with kinase hinge residues^25^. Therefore, compounds that lacked these interactions were removed. Finally, compound poses were visually inspected, and the available compounds were selected for further evaluation.

### Enzymatic assay

The enzymatic assay for assessing RSK2 inhibition was outsourced to Thermo Fisher Scientific, which utilised two highly reliable homogeneous assay platforms: LanthaScreen and Z’-LYTE technologies (https://www.thermofisher.com/z-lyte), both based on Fluorescence Resonance Energy Transfer (FRET). The assay involved co-incubating the test compound with a fluorescein-labelled substrate, kinase, ATP, kinase buffer, and development reagent to initiate the reaction. Following a one-hour incubation period, the reaction was halted by adding a stop solution (EDTA). Fluorescence readings were then obtained using a fluorescence reader. The reported data represent the average of two independent replicates. Additionally, the kinase selectivity of the selected compounds was assessed using Thermo Fisher’s SelectScreen service.

### Cell culture

Human pancreatic cancer cell lines, AsPC-1 (#60494), PANC-1 (#60284), and MIA PaCa-2 (#60139), were obstained from the Bioresource Collection and Research Centre (BCRC, Hsinchu, Taiwan). AsPC-1 cells were cultured in Roswell Park Memorial Institute 1640 (RPMI-1640, Thermo Fisher Scientific, MA, USA), whereas PANC-1 and MIA PaCa-2 cells were maintained in Dulbecco’s modified Eagle’s medium (DMEM, Thermo Fisher Scientific, MA, USA). Both media were supplemented with 10% foetal bovine serum (FBS, v/v), 100 U/mL penicillin, 100 μg/mL streptomycin, and 2.5 μg/mL amphotericin B (Biological Industries Ltd., Kibbutz Beit HaEmek, Israel). The cells were incubated at 37 °C in a humidified atmosphere 5% CO_2_ and 95% air.

### Cell viability

Cell viability was assessed using the MTT (3–(4,5-dimethylthiazol-2-yl)-2,5-diphenyltetrazolium bromide; Sigma-Aldrich, St. Louis, Missouri, USA). Cells were seeded at a density of 5,000 cells per well in a 96-well plate and incubated overnight in standard culture conditions. After treatment with varying concentrations of the test compounds for 72 h. Following this period, the culture medium was aspirated, and 100 μL of MTT solution (0.5 mg/mL in phosphate-buffered saline, PBS) was added to each well. Following incubation at 37 °C for 1 h, the resulting formazan crystals were dissolved in dimethyl sulfoxide (DMSO), and absorbance was measured at 550 nm using an ELISA reader (Beckman Coulter, Brea, CA, USA). The 50% inhibitory concentration (IC_50_) was calculated by comparing the absorbance of treated and control groups.

### Cell proliferation

Cells were seeded at 5,000 cells per well in a 96-well plate and treated with different concentrations of the test compounds for 72 h. After treatment, cells were fixed with 10% trichloroacetic acid (TCA) and washed with phosphate-buffered saline (PBS). The fixed cells were stained with 100 μL of SRB solution (0.4% SRB in 1% acetic acid) for 15 min, followed by three washes with 1% acetic acid. The bound dye was solubilised in 10 mM Tris buffer, and absorbance was measured at 515 nm using an ELISA reader (Beckman Coulter, CA, USA). Another commonly used method for detecting cell proliferation is the Bromodeoxyuridine (BrdU) assay (Millipore, Merck KGaA, Darmstadt, Germany). Cells were seeded at a density of 10,000 cells per well in a 96-well plate and treated with varying concentrations of the test compounds for 72 h. BrdU was then added to the cells for at least 2 h to allow incorporation into proliferating cells. After binding with the anti-BrdU antibody, the absorbance was measured at 450 nm using an ELISA reader. The 50% growth inhibition (GI_50_) was calculated using the formula: 100-[(T_x_-T_0_)/(C-T_0_)] × 100.

### Colony formation assay

To assess colony-forming ability, AsPC-1, MIA PaCa-2, and PANC-1 cells were plated at densities of 3,000 or 5,000 cells per well in 6-well plates and treated with varying concentrations of the test compound or DMSO. After 7–14 days of incubation, cell colonies were fixed and stained with crystal violet for 1 h, followed by three washing steps. The stained colonies were then photographed and quantified using ImageJ software (NIH, Bethesda, Maryland, USA).

### Wound healing assay

To assess cell migration, a wound healing assay was performed using Culture-Inserts 3 Well (ibidi, Gräfelfing, Munich, Germany). A total of 70 μL of cell suspension (1 × 10³ cells/mL) was seeded in each well and allowed to attach for 24 h. Following cell attachment, the Culture-Insert 3 Well was removed, creating two cell-free gaps (each 500 μm in width). Wound closure was monitored at specified time points using an inverted phase-contrast microscope. The percentage of wound closure was quantified using ImageJ software (NIH, Bethesda, Maryland, USA).

### Western blot analysis

Cell lysates were prepared by incubating the cells in lysis buffer (50 mM Tris, 150 mM sodium chloride, 0.1% SDS, 0.5% sodium deoxycholate, and 1% NP-40) at 4 °C for 30 min. The lysates were then centrifuged at 13,000 rpm for 30 min at 4 °C. Protein concentrations were determined using the BCA Protein Assay Kit (Thermo Fisher Scientific, MA, USA). Equal amounts of protein were separated by 10% SDS-polyacrylamide gel electrophoresis (SDS-PAGE) and subsequently transferred onto PVDF membranes. Prior to blocking, the membranes were stained with Ponceau-S solution (Beacle, Inc, Kyoto, Japan) to verify protein transfer. The membranes were incubated overnight at 4 °C with specific primary antibodies, followed by a 1 h incubation with secondary antibodies at room temperature. All primary antibodies were diluted 1:1000 in TBST (Tris-buffered saline with 0.1% Tween 20). Unbound primary antibodies were removed by washing, and the membranes were further incubated with horseradish peroxidase (HRP)-conjugated anti-mouse or anti-rabbit immunoglobulin G (IgG) secondary antibodies diluted 1:5000 in TBST. Protein detection was performed using enhanced chemiluminescence (ECL) reagents (Amersham, Buckinghamshire, UK). Primary antibodies targeting phospho-IκBα (#2859), total IκBα (#4812), and pan-actin (#MAB1501) were purchased from Cell Signalling Technology (Danvers, MA, USA) and Sigma-Aldrich (St. Louis, Missouri, USA), respectively. Secondary antibodies, including goat anti-rabbit IgG (#111–035-003) and goat anti-mouse IgG (#115–035-003), were obtained from Jackson ImmunoResearch (Ely, CB7 4EX, UK).

### Real-time polymerase chain reaction (qPCR)

Total RNA was extracted using the Direct-zol RNA kit (Zymo Research Corporation, CA, USA) following the manufacturer’s protocol. The TRIzol reagent was used to isolate RNA, and 1 μg of messenger RNA (mRNA) was incubated with random primers at 65 °C for 5 min. Complementary DNA (cDNA) was synthesised using the PrimeScript RT Reagent Kit (Takara Bio, Japan) and subsequently analysed by quantitative real-time PCR (qRT-PCR) with SYBR Green PCR Master Mix (Applied Biosystems, MA, USA). Fluorescence signals were detected using the StepOnePlus real-time PCR system (Applied Biosystems, MA, USA). Relative gene expression levels were normalised to 18S rRNA and calculated using the 2^-ΔΔCt^ method. Primer sequences were as follows: human 18S-forward: 5′-AGGCAAAGCAGGAGTCCACTGA-3′, human 18S-reverse: 5′-ATCTGGCGTTCCAGGGACTCAT-3′, human GAPDH forward: 5′-TGGGATTTCCATTGATGACAAG-3′, human GAPDH reverse: 5′-ATTCCACCCATGGCAAATTC-3′, human vimentin-forward: 5′-AACCCGTTGAACCCCATT-3′, human vimentin-reverse: 5′-CCATCCAATCGGTAGTAGC-3′.

### Dataset analyses

RSK2 expression in different pancreatic cancer subtypes was analysed using the Gene Expression Profiling Interactive Analysis 2 (GEPIA2) database, which integrates data from The Cancer Genome Atlas (TCGA) and the Genotype-Tissue Expression (GTEx) project^26^. GEPIA2 facilitates tumour-normal comparisons and utilizes computational methods such as CIBERSORT, EPIC, and quanTIseq to estimate cell-type proportions. Additional analyses include proportion analysis using ANOVA, sub-expression analysis for cell-type-specific differential expression, and survival analysis employing Kaplan-Meier curves and log-rank tests.

The Kaplan-Meier Plotter database was used to assess the association between RSK2 expression and patient survival. This database integrates gene expression and clinical data from the Gene Expression Omnibus (GEO), European Genome-Phenome Archive (EGA), and TCGA, utilising a PostgreSQL server for data management^27^^,^^28^. Prognostic significance was evaluated by stratifying patients based on biomarker expression levels and comparing survival outcomes through Kaplan-Meier plots. Hazard ratios (HRs) and log-rank *p* values were computed to determine statistical significance. To minimise selection bias, all cut-off values within the lower and upper quartiles were tested, and the most statistically significant cut-off (lowest false discovery rate, FDR) was selected. In cases where multiple cut-offs yielded equal significance, the highest HR was chosen.

### Ethical approval and informed consent

This study utilised publicly available online databases for analysis and did not require ethical approval.

### Statistical analysis

All data and statistical analyses were conducted following recommended best practices for experimental design. A minimum of three independent *in vitro* experiments were performed, and results are presented as mean ± standard deviation (SD). GraphPad Prism 10.0 (GraphPad Software, Boston, MA, USA) was used for statistical analyses, with Student’s t-test employed for comparisons between two groups. For animal research data, GraphPad Prism 10.0 and the Student’s t-test were also applied. Statistical significance was set at *p* < 0.05, with the following notation: *p* < 0.05 (*), *p* < 0.01 (**), *p* < 0.001 (***), and *p* < 0.0001 (****).

## Results

### Selection and validation of RSK2 inhibitor

**Figure 1.**
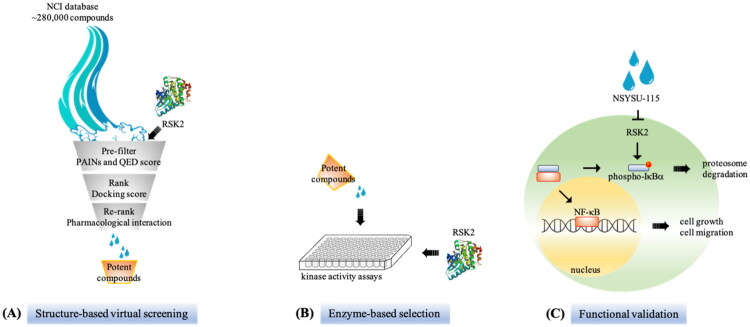
Overview of study. **(A)** NCI (National Cancer Institute) chemical library was used for a virtual screening of RSK2 inhibitors. **(B)** The top-ranked compounds were selected for enzyme-based assays to identify their activity against RSK2. **(C)** The potent inhibitor was further validated for *in vitro* cell growth and migration function.

**Figure 2.**
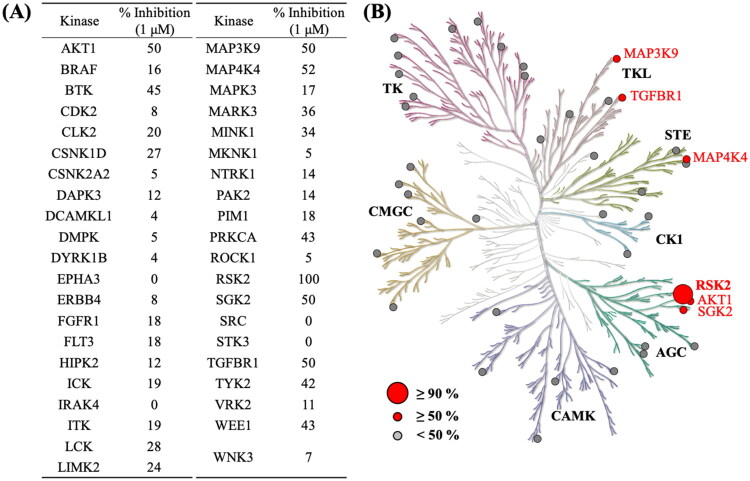
An analysis of the selectivity profile across the kinome. **(A)** NSYSU-115 was tested at 1 μM across a panel of 41 kinases. **(B)** NSYSU-115 is selective towards RSK2. Red dots indicate kinases where the compounds exhibit an inhibition effect greater than 50%.

**Figure 3.**
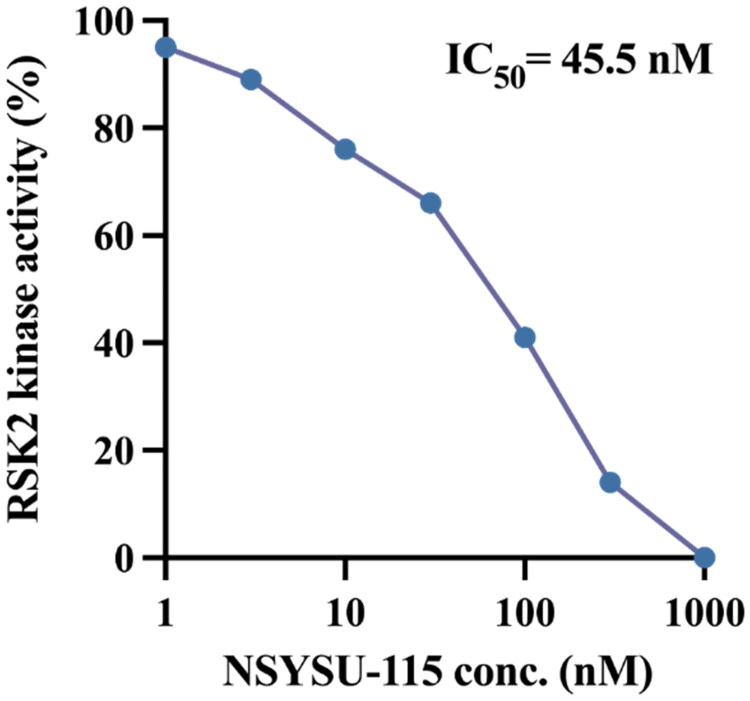
Kinase inhibition of NSYSU-115. The kinase inhibition assay was used to test the efficacy of compounds. The IC_50_ (half maximal inhibitory concentration) of NSYSU-115 on RSK2 kinase activity is 45.5 nM.

Clinical analysis indicates that RSK2 mRNA expression is correlated with clinical outcomes in pancreatic cancer patients (Supplemental Figure 1). Therefore, we conducted a structure-based virtual screening to identify potential RSK2 inhibitors to treat pancreatic cancer (Figure 1). The initial screening involved filtering compounds from the NCI database based on favourable physicochemical drug properties, including the exclusion of compounds containing PAINS structures and those with unfavourable QED scores^19^^,^^29^. The remaining compounds were docked into the RSK2 binding site using Glide^24^ and ranked according to their docking scores. Given that kinase inhibitors typically form hydrogen bonds with hinge residues^25^, top-ranked compounds lacking these interactions with the RSK2 hinge residues were excluded. The remaining potential inhibitors were visually inspected, and available compounds were selected for further validation to confirm their inhibitory activity. In this study, we evaluated the selectivity of the newly identified inhibitors. A panel of 41 kinases from various human kinome families was tested (Figure 2A). Notably, NSYSU-115 exhibited exceptional inhibitory effects on RSK2 (Figure 2B), a serine/threonine kinase and within the p90 ribosomal S6 kinase (RSK) family. NSYSU-115 demonstrated the most potent inhibitory effect on RSK2, with an IC_50_ value of 45.5 nM (Figure 3).

**Figure 4.**
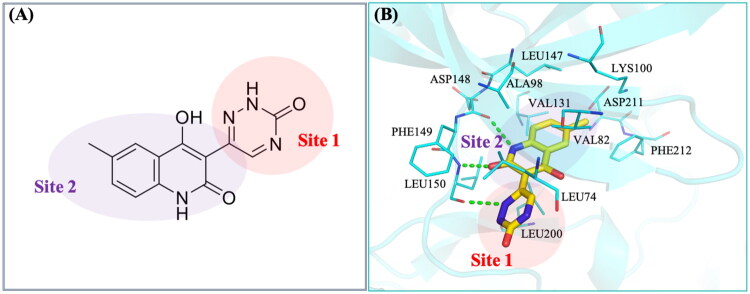
Interactions of NSYSU-115 in RSK2. **(A)** Groups of compound structures in two sites. Quinoline (purple) and triazine (red) groups were filling in two sites of RSK2. **(B)** Docking pose of NSYSU-115. The compound (yellow) was docked into the RSK2 binding site (blue). The binding site residues are shown as stick representations, and hydrogen bonds are illustrated with dashed green lines.

Next, we conducted an interaction analysis of NSYSU-115 to further elucidate its binding mechanism with RSK2 residues (Figure 4). Structurally, NSYSU-115 consists primarily of quinoline and triazine groups (Figure 4A). The quinoline group is positioned within the hinge region, mimicking the adenosine moiety of ATP. It forms two hydrogen bonds with residues ASP148 and LEU150 while engaging in hydrophobic interactions with residues Val82, Ala98, Val131, Leu147, Leu200, and Phe212 (Figure 4B). Additionally, the triazine group forms a hydrogen bond with LEU150 and interacts hydrophobically with Leu74 (Figure 4B). The interaction with Leu74 is hydrophobic; therefore, it is not labelled in the figure. These interactions likely contribute to the potent inhibitory activity of NSYSU-115 against RSK2.

### NSYSU-115 exhibits a significant inhibitory effect on both the viability and proliferation in pancreatic cancer cell lines

**Figure 5.**
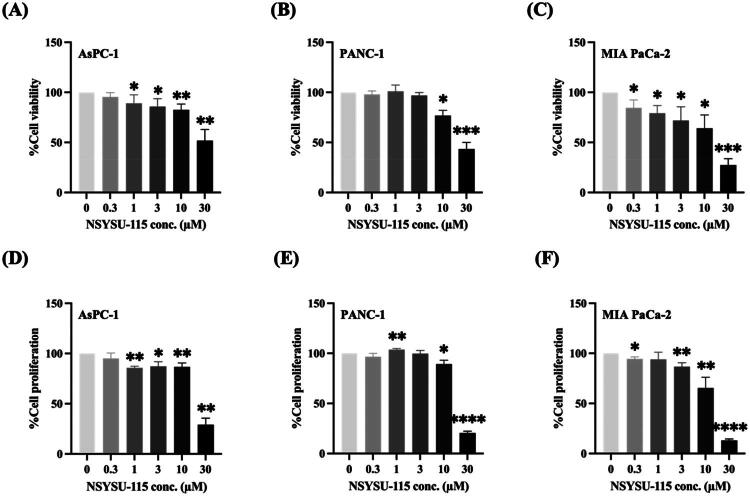
Effects of NSYSU-115 on cell viability and cell proliferation in pancreatic cancer cell lines. Cell viability was assessed using MTT assays in three pancreatic cancer cell lines: AsPC-1 **(A)**, PANC-1 **(B)**, and MIA PaCa-2 **(C)**. Cell proliferation was measured using SRB assays in AsPC-1 **(D)**, PANC-1 **(E)**, and MIA PaCa-2 **(F)**. Cells were treated with either vehicle control (0.1% DMSO) or various concentrations of NSYSU-115 (0.3, 1, 3, 10, and 30 µM) for 72 hours. The data presented, expressed as mean ± SD, are based on a minimum of three independent experiments. Statistical significance is indicated by asterisks: *, *p* < 0.05; **, *p* < 0.01; ***, *p* < 0.001; ****, *p* < 0.0001, compared to the control group.

We conducted experiments using three pancreatic cancer cell lines: AsPC-1 (pancreatic adenocarcinoma), PANC-1 (pancreatic epithelioid carcinoma), and MIA PaCa-2 (pancreatic ductal adenocarcinoma). The AsPC-1 cell line is derived from a metastatic site within the pancreas, while PANC-1 originates from the pancreatic duct, providing a broader representation of pancreatic cancer types. MIA PaCa-2, a well-known human pancreatic ductal adenocarcinoma (PDAC) cell line^30^, consists of both round and spindle-shaped adherent cells, as well as round floating cells. As shown in Figure 5A, treatment with NSYSU-115 resulted in a dose-dependent reduction in cell viability in AsPC-1 cells, with decreases of 11%, 14.3%, 17.4%, and 48.2% at concentrations of 1, 3, 10, and 30 μM, respectively. A similar trend was observed in the reduction of cell proliferation in AsPC-1 cells, with decreases of 14.3%, 13%, 13.3%, and 70.7% at the same respective concentrations (Figure 5D).

In PANC-1 cells, NSYSU-115 significantly reduced cell viability, with decreases of 23.2% and 56.6% at 10 and 30 μM, respectively (Figure 5B). Corresponding reductions in cell proliferation were observed at these concentrations, showing declines of 10.6% and 79.4% (Figure 5E). In MIA PaCa-2 cells, NSYSU-115 also induced a dose-dependent decrease in cell viability, with significant inhibition starting at 0.3 μM. Viability reductions of 15.5%, 20.8%, 28.1%, 35.8%, and 82.6% were observed across increasing concentrations (Figure 5C). Cell proliferation also declined significantly starting at 3 μM, showing inhibition rates of 13.3%, 34.6%, and 87.0% (Figure 5F). Supplementary BrdU assays further confirmed these results, demonstrating proliferation inhibition of 70.15% and 92.6% at 10 and 30 μM in PANC-1 cells (Supplementary Figure 3A), with a similar pattern in MIA PaCa-2 cells (Supplementary Figure 3B).

Both the MTT assay and the SRB assay confirmed a substantial decrease in cell viability and proliferation at the highest concentration tested (30 μM) of NSYSU-115 (Figure 5). However, significant effects were observed only in MIA PaCa-2 cells at the lowest concentration (0.3 μM), indicating that certain cell types, such as PDAC, exhibit greater sensitivity to the NSYSU-115. Further calculations using relevant formulas and software determined that the GI_50_ (growth inhibition of 50%) and IC_50_ (half-maximal inhibitory concentration) values for AsPC-1 were 22.7 μM and 31.2 μM, respectively, while for PANC-1, these values were 21.4 μM and 20.1 μM. Additionally, for MIA PaCa-2 cells, these values were 15.9 μM and 17.7 μM, respectively. Additionally, BrdU assay-based proliferation measurements yielded lower GI_50_ values of 9.15 μM for PANC-1 and 8.15 μM for MIA PaCa-2 cells, further supporting the antiproliferative potential of NSYSU-115. These results suggest that NSYSU-115 exerts a stronger inhibitory effect on cell proliferation than on cell viability in pancreatic cancer cell lines, highlighting its potential as a selective growth inhibitor.

### NSYSU-115 demonstrates a dose-dependent inhibitory effect on colony formation in the pancreatic cancer cell lines

**Figure 6.**
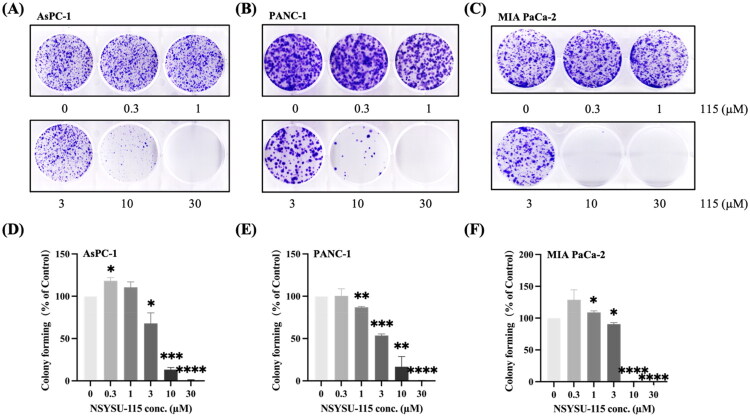
Effects of NSYSU-115 on colony-forming ability of pancreatic cancer cell lines. Cells were treated with either vehicle control (0.1% DMSO) or various concentrations of NSYSU-115 (0.3, 1, 3, 10, and 30 µM) for 7 days. Crystal violet was used to stain colonies for enhanced visibility, and the number of colonies was quantified using ImageJ software. The results in **(A)** and **(D)** were obtained from AsPC-1, whereas the results in **(B)** and **(E)** were obtained from PANC-1. Additionally, the results in **(C)** and **(F)** were generated from MIA PaCa-2. The presented data, representing the mean ± SD, are derived from a minimum of three independent experiments. Statistical significance is denoted by asterisks: *, *p <* 0.05; **, *p <* 0.01; ***, *p <* 0.001; ****, *p <* 0.0001 compared to the control group.

The clonogenic assay, also known as the colony formation assay, is an *in vitro* method used to evaluate the ability of a single cell to proliferate and form a colony^31^. It is a preferred technique for determining cell reproductive death following exposure to ionising radiation and can also be applied to assess the efficacy of various cytotoxic agents. In the AsPC-1 pancreatic cancer cell line, treatment with NSYSU-115 at concentrations of 3, 10, and 30 μM results in a progressive decrease in forming colonies (Figure 6A), consistent with the observed reductions of 32.1%, 86.7%, and 99.2%, respectively (Figure 6D). Similarly, in the PANC-1 pancreatic cancer cell line, NSYSU-115 exhibits a dose-dependent inhibitory effect on colony formation across concentrations ranging from 1 to 30 μM, leading to survival reductions between 13.2% and 99.6% (Figure 6B and E). In the MIA PaCa-2 cell line, treatment with low concentrations of NSYSU-115 led to an increase in colony formation, particularly a significant 9% rise at 1 µM. Conversely, treatment with 3 µM NSYSU-115 resulted in a notable 9.5% reduction in colony formation, a response similar to that observed in AsPC-1 cells treated with NSYSU-115. Unlike the other two pancreatic cancer cell lines, MIA PaCa-2 cells exhibited complete inhibition of colony formation at higher concentrations of 10 and 30 µM (Figure 6C and F). These findings indicate that MIA PaCa-2 is more sensitive to NSYSU-115 compared to the other two cell lines. In conclusion, NSYSU-115 significantly inhibits cell reproductive capacity in a dose-dependent manner in pancreatic cancer cells, highlighting its potential as an effective cytotoxic agent.

### NSYSU-115 inhibits phosphorylation and degradation downstream of RSK2 in pancreatic cancer cell lines

RSK2 plays a critical role in various cellular processes, including the regulation of the cell cycle, cytoskeletal organisation, and cell survival^32^. Upon activation by specific stimuli, RSK2 directly phosphorylates IκBα (inhibitor of NF-κB), primarily at the Ser-32 and Ser-36 residues, which triggers its ubiquitination and subsequent degradation^18^^,^^33^. Notably, RSK2 specifically targets the Ser-32 site. Once degraded, IκBα releases NF-κB, allowing it to translocate from the cytoplasm into the nucleus, where it activates the transcription of genes involved in cell survival and proliferation^34–36^. The ratio of phosphorylated IκBα to total IκBα is therefore commonly used as an indicator of NF-κB pathway activation.

**Figure 7.**
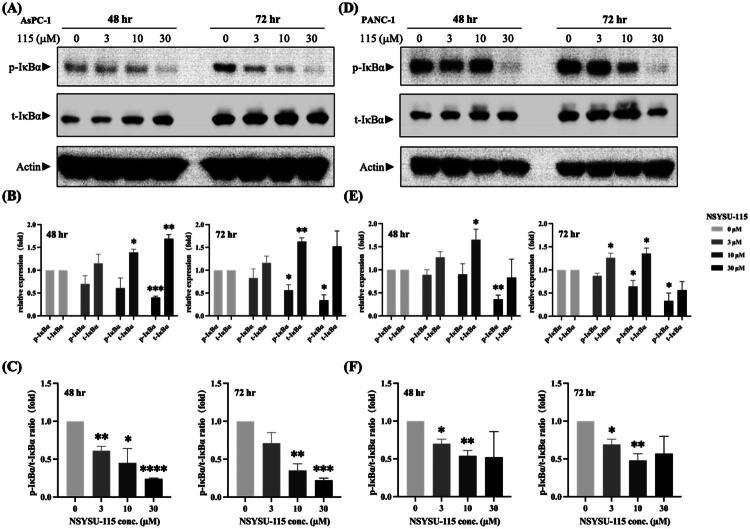
Inhibitory effects of NSYSU-115 on RSK2 downstream phosphorylation and degradation. AsPC-1 **(A)** and PANC-1 **(D)** cells were treated with either vehicle control (0.1% DMSO) or NSYSU-115 at concentrations of 3, 10, and 30 μM for 48 and 72 hours. Following treatment, cell lysates were subjected to Western blot analysis using specific antibodies. Band intensities were quantified using ImageJ software and normalised to the loading control for AsPC-1 **(B)** and PANC-1 **(E)**. The ratios of phosphorylated IκBα (p-IκBα) to total IκBα (t-IκBα) were calculated based on the quantitative results and shown for AsPC-1 **(C)** and PANC-1 **(F)**. The presented data, reflecting the mean ± SD, were obtained from a minimum of three independent experiments. Statistical significance is indicated by asterisks: *, *p <* 0.05; **, *p <* 0.01; ***, *p <* 0.001; ****, *p <* 0.0001, in comparison to the control group.

After treating pancreatic cancer cells with NSYSU-115 for 48 h, a significant reduction in the phosphorylation of IκBα is observed, particularly at a concentration of 30 μM. This reduction was consistent in both the AsPC-1 (Figure 7A) and PANC-1 (Figure 7D) cell. After 72 h of NSYSU-115 treatment, a phosphorylated IκBα levels continued to decrease at 10 and 30 μM, as confirmed by quantitative analysis (Figure 7B and E), indicating a time- and dose-dependent inhibition of RSK2-mediated phosphorylation. Regarding total IκBα protein levels, AsPC-1 cells exhibited a significant increase following treatment with 10 μM NSYSU-115 at both 48 and 72 h, with the strongest upregulation seen at 30 μM after 48 h (Figure 7B). In PANC-1 cells, a similar trend was observed, though the increase in total IκBα was limited to 72-h treatments at 3 and 10 μM (Figure 7E). To further assess NF-κB signalling, we analysed the ratio of phosphorylated IκBα to total IκBα. In AsPC-1 cells, NSYSU-115 induced a significant, dose-dependent reduction in this ratio at both 48 and 72 h (Figure 7C). A similar inhibitory pattern was observed in PANC-1 cells, though it occurred at lower concentrations (Figure 7F). Collectively, these results suggest that NSYSU-115 suppresses NF-κB signalling by inhibiting RSK2-mediated phosphorylation of IκBα, thereby preventing its degradation and stabilising IκBα protein levels.

### NSYSU-115 demonstrates an inhibitory effect on migration in the pancreatic cancer cell lines

**Figure 8.**
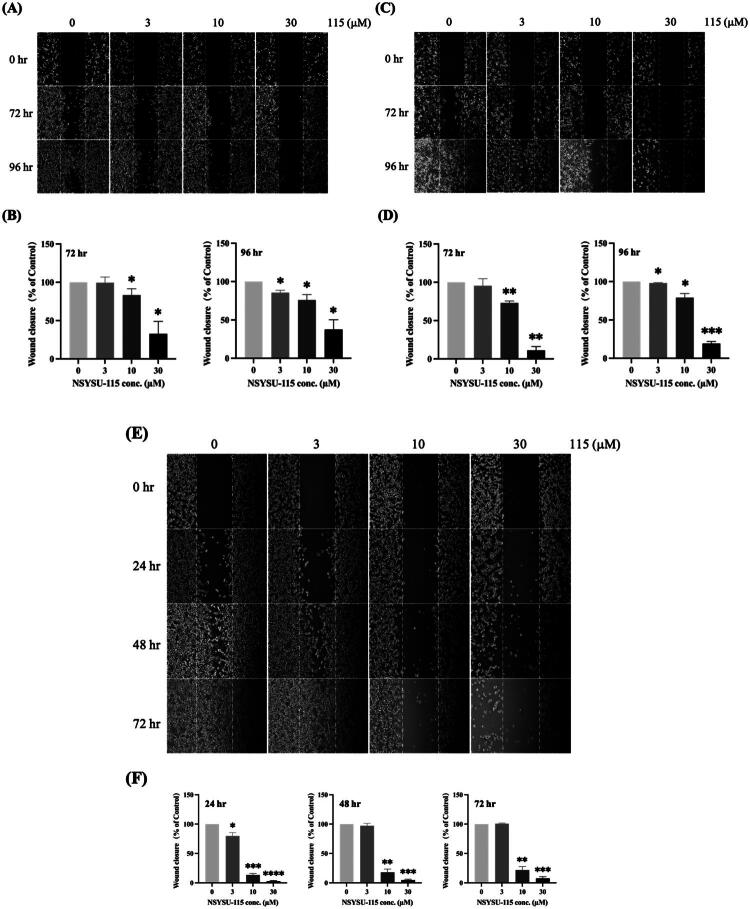
Inhibitory effects of NSYSU-115 on cell migration in pancreatic cancer cell lines. Cells, including AsPC-1 **(A)**, PANC-1 **(C)**, and MIA PaCa-2 **(E), **were treated with either vehicle control (0.1% DMSO) or various concentrations of NSYSU-115 (3, 10, and 30 µM) for the indicated time points. A wound distance of 500 µm was marked with white dashed lines, and images were captured using an inverted phase-contrast microscope. For each condition, at least 5 images were randomly selected , and the wound distance was measured using ImageJ software. The quantification results for AsPC-1 **(B)** at 72 hours are shown on the left, and at 96 hours on the right. Similarly, the quantification results for PANC-1 **(D)** at 72 hours are displayed on the left, and at 96 hours on the right. For MIA PaCa-2 **(F)**, the quantification results at 24, 48, and 72 hours are displayed at the bottom. Data are presented as the mean ± SD, based on a minimum of three independent experiments. Statistical significance is denoted by asterisks: *, *p* < 0.05; **, *p* < 0.01; ***, *p* < 0.001; ****, *p* < 0.0001, compared to the control group.

Previous research has shown that RSK plays a role in promoting EMT, which contributes to increased cell migration and invasion^13^. Specifically, RSK2 activation in pancreatic cancer cell lines has been associated with EMT, enhancing the motility and invasive behaviour of cancer cells^37^. Our experimental findings indicate that inhibiting cell migration in AsPC-1 cells is significantly influenced by both the concentration and duration of NSYSU-115 treatment (Figure 8A). A similar pattern was observed in PANC-1 cells (Figure 8C). Quantitative analysis showed that after 72 h of treatment, NSYSU-115 significantly reduced the migration ability of AsPC-1 cells to 83.4% and 32.7% at concentrations of 10 and 30 µM, respectively (Figure 8B left). After 96 h, the migration ability further decreased to 85.6%, 75.9%, and 37.6% at concentrations of 3, 10, and 30 µM, respectively (Figure 8B right). Comparable results were obtained in PANC-1 cells, where NSYSU-115 significantly reduced migration ability to 73.1% at 10 µM and 11.1% at 30 µM after 72 h. At 96 h, migration was further inhibited to 98.0%, 79.0%, and 19.2% at concentrations of 3, 10, and 30 µM, respectively (Figure 8D). For MIA PaCa-2 cells, treatment with 3 µM NSYSU-115 resulted in a significant approximately 20.2% reduction in cell migration ability (Figure 8E and F left). Compared to the other two pancreatic cancer cell lines, NSYSU-115 effectively decreased cell migration at lower concentrations and shorter treatment durations. Additionally, treatments with 10 µM and 30 µM NSYSU-115 consistently reduced cell migration by approximately 80% and 95%, respectively, at all time points (Figure 8F). Unlike the other two cell lines, MIA PaCa-2 cells exhibited significant inhibition of cell migration with only a 10 µM concentration of NSYSU-115 (Figure 8B, D, and F). In conclusion, NSYSU-115 exhibits a dose- and time-dependent inhibitory effect on the migration ability of pancreatic cancer cell lines, demonstrating its potential to suppress cancer cell motility and invasiveness.

### NSYSU-115 exhibits a significant inhibitory effect on vimentin expression in pancreatic cancer cell lines

**Figure 9.**
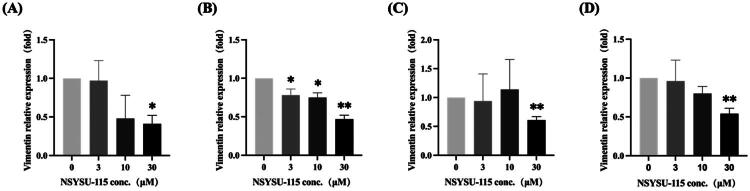
Inhibitory effects of NSYSU-115 on vimentin expression in pancreatic cancer cell lines. Cells were treated with either vehicle control (0.1% DMSO) or various concentrations of NSYSU-115 (3, 10, and 30 µM). RT-qPCR analysis was performed to quantify the expression levels of the mesenchymal marker vimentin, using 18S and GAPDH as internal controls. AsPC-1 cells were treated for 48 hours **(A)** and 72 hours **(C)**, with 18S as the internal control. PANC-1 cells were treated for 48 hours **(B)** and 72 hours **(D)**, with GAPDH as the internal control. Data are presented as the mean ± SD, based on a minimum of three independent experiments. Statistical significance is indicated by asterisks: *, *p* < 0.05; **, *p* < 0.01, compared to the control group.

Previous studies suggest that NSYSU-115 may slow down the progression of EMT by inhibiting RSK2, a key regulator of EMT, with RSK2 and NF-κB playing critical roles in this process. At a concentration of 30 µM, NSYSU-115 consistently reduced vimentin expression by half across various pancreatic cancer cell lines, regardless of the treatment duration (Figure 9). In contrast, lower concentrations (3 and 10 µM) significantly inhibited vimentin expression only in PANC-1 cells after 48 h of treatment (Figure 9B). Overall, NSYSU-115 effectively reduces vimentin expression in pancreatic cancer cells at higher doses. Furthermore, as shown in Figure 8, NSYSU-115 also suppresses EMT, thereby limiting cell migration.

## Discussion

RSK2 levels are elevated in various cancers, including acute myeloid leukaemia, glioblastoma multiforme, stomach adenocarcinoma^38^, and pancreatic adenocarcinoma (Supplemental Figure 1). Clinical studies indicate a correlation between high RSK2 expression and poor survival outcomes in pancreatic ductal adenocarcinoma (Supplemental Figure 1D and E). To explore this relationship, we performed structure-based virtual screening to identify a novel RSK2 inhibitor (Figure 1). The compound NSYSU-115 exhibited an IC_50_ of 45.5 nM against RSK2. In vitro assays demonstrated dose- and time-dependent inhibition of RSK2 activity, particularly through the reduction of IκBα phosphorylation. NSYSU-115 significantly impaired cell viability, proliferation, and survival while also inhibiting migration and altering EMT marker expression. These findings underscore the role of RSK2 in pancreatic cancer metastasis and highlight its potential as a therapeutic target.

Approximately 80% of pancreatic cancer cases are classified as pancreatic adenocarcinoma, predominantly pancreatic ductal adenocarcinoma (PDAC) ^39^. In pancreatic adenocarcinoma patients, RSK2 mRNA expression is significantly higher than in normal tissues (Supplemental Figure 1A). Molecular and prognostic analyses further classify PDAC into basal and classical subtypes, with the basal subtype exhibiting more aggressive behaviour and poorer prognosis^8^. Both subtypes display significantly elevated RSK2 expression compared to normal tissues (Supplemental Figure 1B), with no substantial differences in RSK2 levels between the two subtypes (Supplemental Figure 1C). These findings indicate that RSK2 is overexpressed in PDAC regardless of subtype, suggesting that its expression is independent of classification.

While the GEPIA2-based survival analysis using a median cut-off did not reach statistical significance, a trend towards poorer survival in the RSK2-high group was observed. To further support our findings, we also consulted the Kaplan-Meier Plotter database, which includes a larger cohort of 1,144 pancreatic cancer patients. Although this dataset does not provide matched normal tissue for expression comparison, it demonstrates that RSK2 expression can vary by up to four-fold between high and low expression groups. Patients with a higher RSK2 expression exhibit a higher risk of mortality, with a median survival reduction of approximately three months and a hazard ratio of 1.2 (Supplemental Figure 1D). Specifically, in pancreatic ductal adenocarcinoma, RSK2 expression is nearly three times higher than in normal tissues, resulting in a median survival decrease of 3.8 months and a hazard ratio of 1.28 (Supplemental Figure 1E). This underscores a significant correlation between RSK2 expression levels and the survival duration of pancreatic cancer patients. These data further confirm the strong correlation between RSK2 expression and patient survival.

Heterogeneity exists not only between individuals but also within tumour cells. Intratumoral heterogeneity contributes to diverse genotypic and phenotypic variations, affecting drug treatment efficacy^7^. Previous studies have classified pancreatic tumours based on transcriptome analysis, including a “virtual dissection strategy” using RNA profiling^8^. Initially, PDAC was categorised into classical and basal-like subtypes, with a hybrid subtype later proposed^40^. Recent studies suggest that multiple subtypes may coexist within the same tumour^41^. The COMPASS trial demonstrated that PDAC patients with the classical subtype responded better to first-line 5-fluorouracil-based regimens than those with the basal-like subtype^42^. As RSK2 expression is consistently elevated in PDAC regardless of subtype (Supplemental Figure 1C), NSYSU-115 may provide therapeutic benefits irrespective of tumour classification.

Previous studies have confirmed that RSK2, activated by hypoxia or ERK signalling, regulates cancer cell movement and invasion through multiple pathways^17^. This includes the transcriptional activation of genes associated with EMT, modulation of integrin activity, and reorganisation of the actin cytoskeleton. Mechanistically, RSK2 phosphorylates IκBα, facilitating NF-κB entry into the nucleus and regulating downstream gene expression. This process induces cytokine secretion, enhancing cancer cell migration and invasion^18^. Additionally, NF-κB, a well-known master regulator, not only promotes malignant transformation through sustained pro-survival signalling and reduced apoptosis but also drives and maintains invasive phenotypes linked to EMT and metastasis^43^. Research shows that NF-κB interacts with EMT-related transcription factors such as Snail, Slug, Twist, Zeb1, and Zeb2, disrupting cell-cell adhesion, remodelling the cytoskeleton, altering cell polarity, and increasing cell motility^43^. These actions collectively enhance cancer cell invasiveness. Based on the discovery of the critical regulatory role of the RSK2-IκBα-NF-κB signalling axis^44^, this study proposes that a leading RSK2 inhibitor holds significant potential for managing pancreatic cancer metastasis (Figures 7 and 8).

RSK2, in addition to NF-κB, is closely linked to EMT and migration^45^. Previous studies have demonstrated that RSK2 interacts with small GTPases, such as RhoA, to promote cytoskeletal reorganisation, thereby enhancing the migratory capacity of cancer cells^46^. Furthermore, RSK2 stimulates the expression of matrix metalloproteinases (MMPs), leading to extracellular matrix degradation and further facilitating cell invasion and migration^47^. RSK2 activation is significantly associated with the metastatic potential of tumours, making it an important target for cancer metastasis treatment^45^. Therefore, we suggested that NSYSU-115 might not only inhibit EMT and migration by targeting the RSK2-IκBα-NF-κB signalling axis but also block other RSK2-related pathways, achieving a dual inhibitory effect.

The pathogenesis of pancreatic ductal adenocarcinoma (PDAC) involves diverse and overlapping signalling pathways. Although NSYSU-115 was designed as an RSK2 inhibitor, kinase profiling revealed that it also inhibits other kinases, including AKT1, TGFBR1, and MAP4K4 (Figure 2). Thus, the biological effects observed at 30 μM may reflect combined pathway modulation rather than RSK2 inhibition alone. However, at lower concentrations (1–10 μM), NSYSU-115 significantly suppressed colony formation, migration, and NF-κB activity (Figures 6–8) with minimal expected inhibition of other kinases. These findings suggest that RSK2 inhibition plays a more dominant role at lower doses, supporting a dose-dependent and selective mechanism of action.

To determine whether NSYSU-115 has broader applicability, gene expression and overall survival curve were performed for patient samples spanning 33 different cancer types, forming a survival map (Supplemental Figure 2A). From these data, five cancers exhibiting the same trend as pancreatic cancer were identified: ACC (adrenocortical carcinoma), DLBC (lymphoid neoplasm diffuse large B-cell lymphoma), ESCA (esophageal carcinoma), KICH (kidney chromophobe), and LGG (brain lower grade glioma) (Supplemental Figure 2B). In specimens from these six cancer types, with hazard ratio (HR), higher expression of RSK2 is associated with shorter survival time. Higher expression of NF-κB is corresponding with shorter survival time or no significant effect. By contrast, higher IκBα expression is associated with longer survival time, possibly because IκBα binds NF-κB and prevents cancer cells from acquiring malignant traits. In conclusion, beyond pancreatic cancer, NSYSU-115 may also hold promise as a therapeutic candidate for five other cancer types in the future.

Several RSK2 inhibitors have been identified, each with distinct mechanisms of action, structural features, and inhibitory potencies (IC_50_ values). BI-D1870 targets the ATP-binding site in the N-terminal kinase domain (NTKD) with an IC_50_ of 10–30 nM^48^. SL0101, a kaempferol glycoside, also binds to the NTKD with an IC_50_ of 89 nM^49^. LJH685 selectively inhibits RSK2 at nanomolar concentrations^50^, while FMK inhibitors irreversibly modify a cysteine residue with IC_50_ values in the micromolar range^51^. BRD7389 acts as an allosteric inhibitor with an IC_50_ of 1–5 μM^52^. PMD-026, the first RSK inhibitor in clinical trials, is being evaluated in a phase 1/1b study for metastatic triple-negative breast cancer (NCT04115306) ^32^. Structural analysis suggests that NSYSU-115 primarily interacts with the first 212 amino acids of RSK2 (Figure 4B), forming hydrogen bonds with Asp148 and Leu150 in the NTKD, similar to known RSK2 inhibitors^38^. Its mechanism of inhibition may be similar to that of known inhibitors like BI-D1870, SL0101, and LJH685, with comparable IC_50_ values. Notably, previous studies have shown that RSK2 inhibitors with low nanomolar potency can effectively reduce tumour growth in xenograft models without significant toxicity^53^. Based on this, we plan to further evaluate the therapeutic potential, pharmacokinetic properties, and antitumor efficacy of NSYSU-115 in pancreatic cancer xenograft models in future studies.

This study highlights the need for targeted therapies in pancreatic cancer, demonstrating that RSK2 overexpression correlates with poor survival. Currently, no RSK2 inhibitors have been tested in clinical trials for pancreatic cancer. Through structure-based virtual screening, NSYSU-115 was identified as a novel RSK2 inhibitor that effectively reduces cell colony formation, cell viability, proliferation, and migration while decreasing IκBα phosphorylation and EMT marker expression.

These findings support further investigation of NSYSU-115 as a potential therapeutic agent for pancreatic cancer.

## Supplementary Material

20250515_Supplemental_information_Final_ Clean.docx

## Data Availability

The datasets used in this study are available from the corresponding author upon request.
