# Supplementary material for: Discovery of a novel RSK2 inhibitor for the treatment of metastatic pancreatic cancer
Source: J Enzyme Inhib Med Chem. 2025 Aug 5;40(1):2538673. doi: 10.1080/14756366.2025.2538673 (PMC12326382; doi:10.1080/14756366.2025.2538673)
Supplement: 20250515_Supplemental_information_Final_ Clean.docx [file IENZ_A_2538673_SM3723.docx]

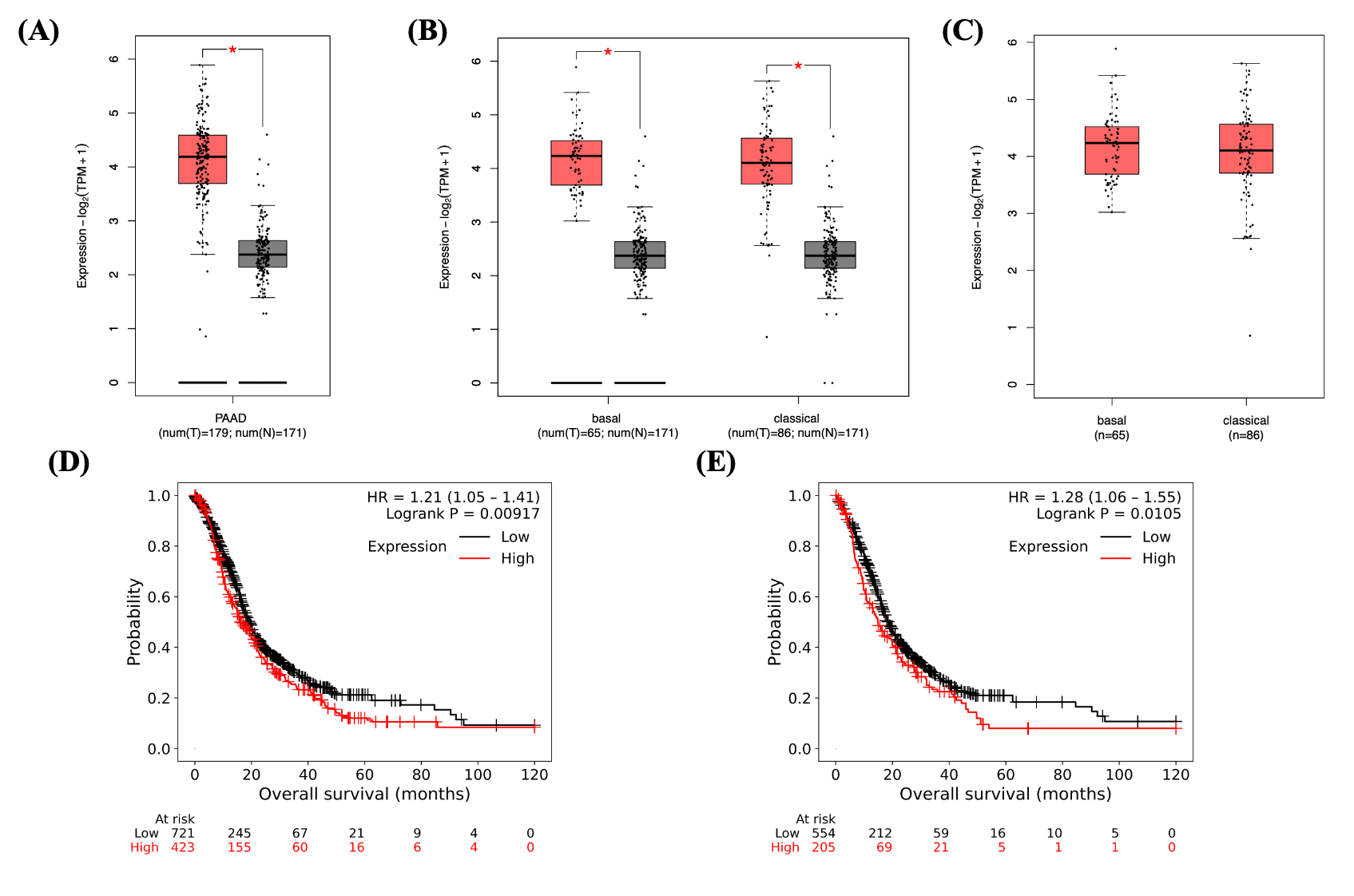
**Supplemental Figure 1. RSK2 mRNA expression correlates with patient survival in pancreatic cancer.**

**(A)** Box plots demonstrate the upregulation of RSK2 in pancreatic adenocarcinoma (PAAD), where tumor samples are depicted in red and normal samples in gray. The height of each bar represents the median expression level in specific tumor or normal tissues (T=Tumor, N=Normal). **(B)** PAAD subtypes, comprising basal and classical to normal, display elevated expression levels of RSK2. **(C)** PAAD subtypes, comprising basal to classical, display equivalent expression levels of RSK2 The data were analyzed utilizing the Gene Expression Profiling Interactive Analysis 2 (GEPIA2) database. *, *p* < 0.05. **(D)** Kaplan-Meier overall survival (OS) curves were created with Kaplan-Meier Plotter, comparing patients across grades I-IV of pancreatic cancer collectively. Individuals with elevated RSK2 expression levels displayed shorter survival times. **(E)** A prevalent subtype of pancreatic cancer, pancreatic ductal adenocarcinoma (PDAC), representing a substantial proportion, also showed a parallel trend. Statistical significance was determined using the Log-rank test.


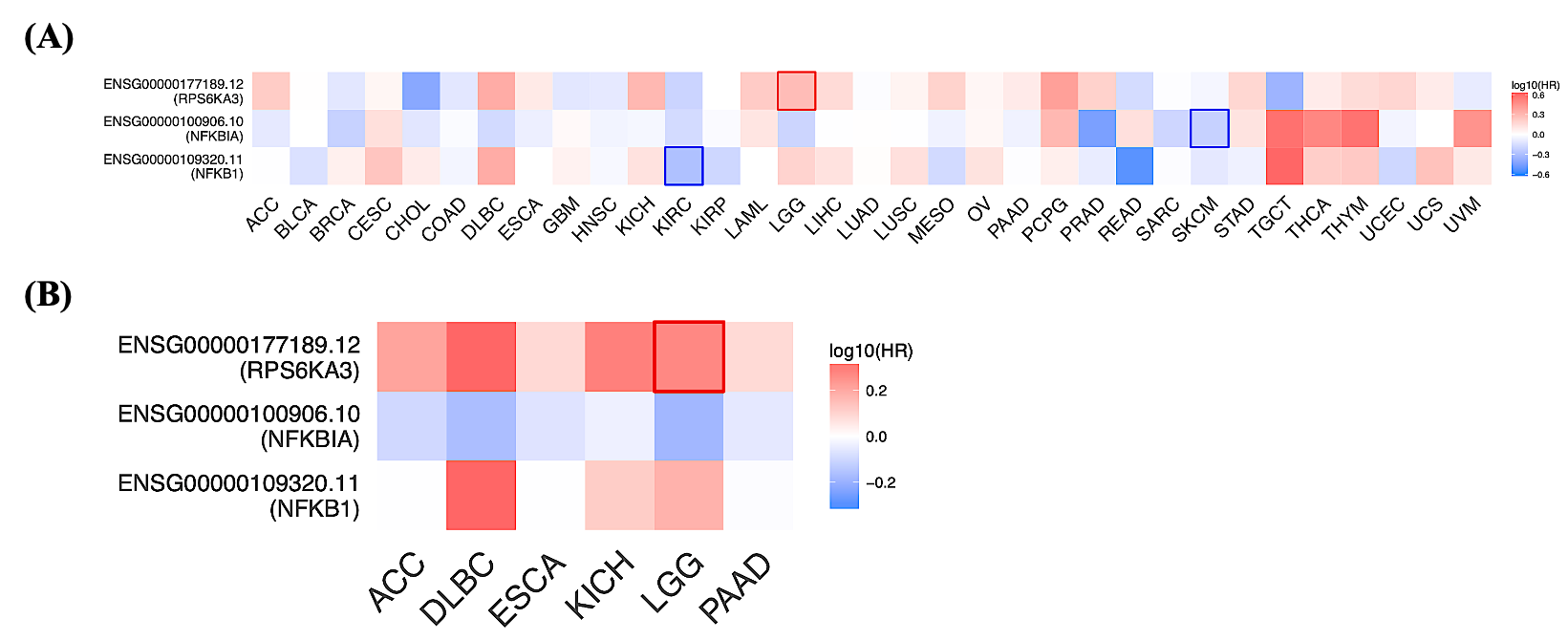
**Supplemental Figure 2. Survival map with different gene expressions.**

The survival map was generated using overall survival curves, with p-values adjusted by the FDR method. This map includes all cancer types **(A)** as well as the cancer types that exhibit similar trends **(B)**. RSK2 (RPS6KA3), IκBα (NKFBIA), and NF-κB (NFKB1) are annotated with different names. The data were analyzed using the Gene Expression Profiling Interactive Analysis 2 (GEPIA2) database. *, *p* < 0.05.


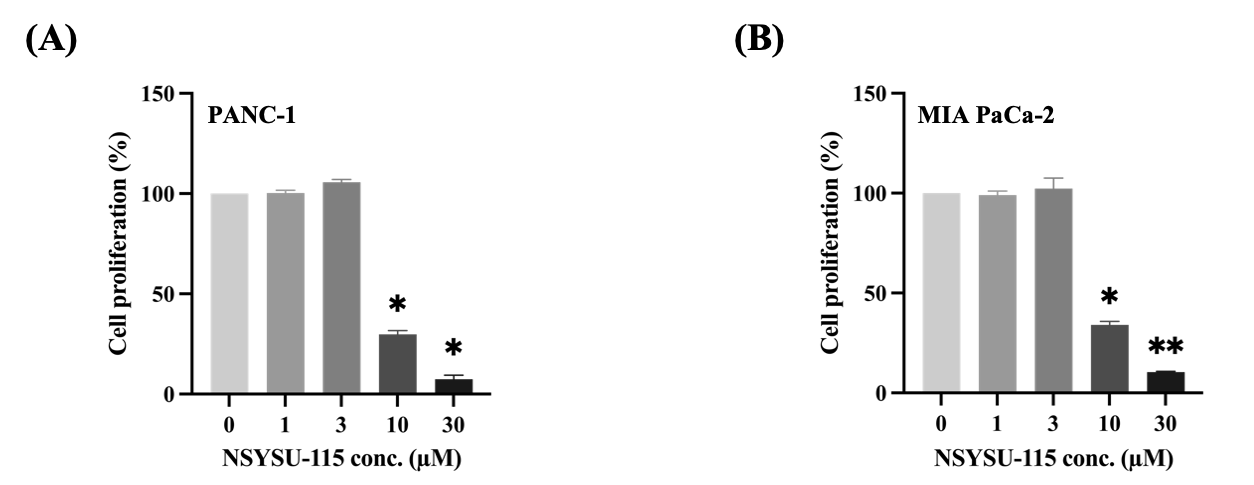
**Supplemental Figure 3. Effects of NSYSU-115 on cell proliferation in pancreatic cancer cell lines.**

Cell proliferation was assessed using BrdU assays in two pancreatic cancer cell lines: PANC-1 **(A)** and MIA PaCa-2 **(B)**. Cells were treated with either vehicle control (0.1% DMSO) or various concentrations of NSYSU-115 (1, 3, 10, and 30 μM) for 72 hours. The data presented, expressed as mean ± SD, are based on a minimum of two independent experiments. Statistical significance is indicated by asterisks: *, *p* < 0.05; **, *p* < 0.01, compared to the control group.
